# Coping with Commitment: Projected Thermal Stress on Coral Reefs under Different Future Scenarios

**DOI:** 10.1371/journal.pone.0005712

**Published:** 2009-06-03

**Authors:** Simon D. Donner

**Affiliations:** Department of Geography, University of British Columbia, Vancouver, Canada; Northeastern University, United States of America

## Abstract

**Background:**

Periods of anomalously warm ocean temperatures can lead to mass coral bleaching. Past studies have concluded that anthropogenic climate change may rapidly increase the frequency of these thermal stress events, leading to declines in coral cover, shifts in the composition of corals and other reef-dwelling organisms, and stress on the human populations who depend on coral reef ecosystems for food, income and shoreline protection. The ability of greenhouse gas mitigation to alter the near-term forecast for coral reefs is limited by the time lag between greenhouse gas emissions and the physical climate response.

**Methodology/Principal Findings:**

This study uses observed sea surface temperatures and the results of global climate model forced with five different future emissions scenarios to evaluate the “committed warming” for coral reefs worldwide. The results show that the physical warming commitment from current accumulation of greenhouse gases in the atmosphere could cause over half of the world's coral reefs to experience harmfully frequent (p≥0.2 year^−1^) thermal stress by 2080. An additional “societal” warming commitment, caused by the time required to shift from a business-as-usual emissions trajectory to a 550 ppm CO_2_ stabilization trajectory, may cause over 80% of the world's coral reefs to experience harmfully frequent events by 2030. Thermal adaptation of 1.5°C would delay the thermal stress forecast by 50–80 years.

**Conclusions/Significance:**

The results suggest that adaptation – via biological mechanisms, coral community shifts and/or management interventions – could provide time to change the trajectory of greenhouse gas emissions and possibly avoid the recurrence of harmfully frequent events at the majority (97%) of the world's coral reefs this century. Without any thermal adaptation, atmospheric CO_2_ concentrations may need to be stabilized below current levels to avoid the degradation of coral reef ecosystems from frequent thermal stress events.

## Introduction

Anthropogenic climate change threatens the function of coral reef ecosystems and the millions of people across the tropics depending on those ecosystems for food, income and shoreline protection [1]–[4]. Approximately one quarter of the carbon dioxide emitted by human activity is absorbed by the oceans. The rise in oceanic carbon dioxide threatens to reduce rates of calcification by corals and other reef organisms and could eventually limit reef accretion [4], [5]. In addition, ocean temperatures of 1–2°C greater than the usual summer maximum can cause mass coral bleaching, a paling of the reef-building animals caused by a breakdown of the symbiosis with the colourful dinoflagellates *Symbiodinium* [1], [6]. Episodes of mass coral bleaching have led to coral mortality, declines in coral cover and shifts in the population of other reef-dwelling organisms [1], [2], [4], [7]–[11].

The degradation of coral reefs due to ocean warming is expected to progress faster than many other prominently researched impacts of climate change, including ice sheet melting, Amazonian forest dieback, migration of tropical diseases and declines in agricultural productivity [12, see Fig SPM.2]. Anthropogenic forcing is likely (>90%) to have already played a role in recent mass bleaching events, including the 2005 event in the Eastern Caribbean [13]. Past analyses have concluded that ocean warming over the next three to four decades may make mass coral bleaching a frequent occurrence worldwide, depending on assumptions about thermal adaptation [1], [13]–[15]. The marine science and conservation community has responded with calls to stabilize greenhouse gas emissions at a level which would such avoid frequent, severe mass bleaching events [3], [4], [16].

The extent to which greenhouse gas mitigation can alter the long-term forecast for coral reefs is limited by the time lag between greenhouse gas emissions and the physical climate response. The thermal inertia of the deep ocean and other components of the climate system delay the physical response to changes in external forcings like greenhouse gases. This physical commitment is calculated by the difference between the transient response and the equilibrium response of an atmosphere-ocean general circulation model to a change in atmospheric greenhouse gas concentrations. In addition to this physical commitment, the expected lag between a decision to reduce greenhouse gas emissions and the implementation of mitigation activities imparts a societal warming commitment. For example, the slow turnover of capital stock in the energy industry is likely to preclude a very rapid shift from carbon-intensive forms of power generation like conventional coal to alternative options like solar, wind or coal with carbon capture and storage.

**Figure 1:**
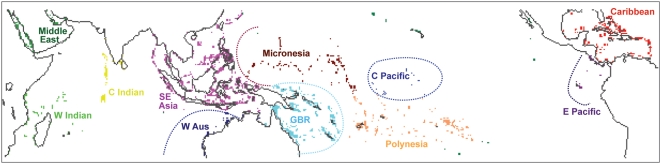
Map of the 0.5°×0.5° coral reef “cells” grouped into eleven ocean provinces. The map depicts the 1687 cells which contain warm-water coral reefs and are described as ocean in the CM2.0 and CM2.1.

**Figure 2:**
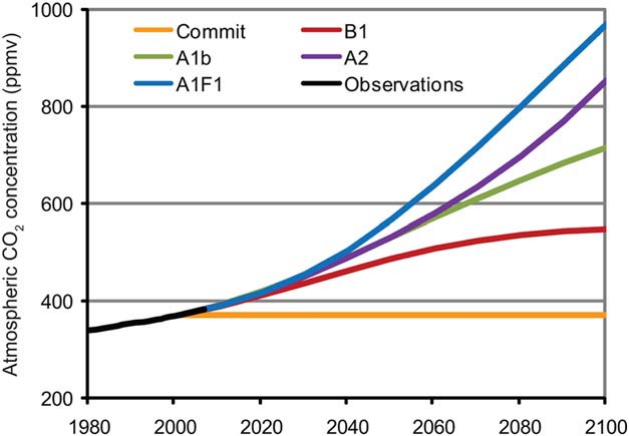
Annual globally averaged atmospheric carbon dioxide concentration (in ppm) from 2000 to 2100 in the five future scenarios considered in this study. The observed global mean concentration from 1980 to 2007 is displayed for comparison. The concentration stabilizes at 370 ppm in the year 2000 in the Commit scenario. The concentration reaches at 550 ppm and 700 ppm by the year 2100, in the B1 and A1b scenarios respectively.

This study specifically examines the implications of “committed warming” for coral reef ecosystems worldwide (Fig. 1) for the first time. The likelihood of severe mass coral bleaching occurring at reefs worldwide over the next two centuries is estimated by integrated satellite-observations of sea surface temperatures and thermal stress indices with output from simulations of the Geophysical Fluid Dynamics Laboratory (GFDL) climate models CM2.0 and CM2.1 [17]. The analysis is conducted for five different future scenarios including i) the stabilization of atmospheric greenhouse gas concentrations at year 2000 levels (“Commit”), ii) the SRES B1 mitigation scenario, in which atmospheric CO_2_ concentrations could stabilize at 550 ppm in the year 2100, and iii) the SRES A1b “business-as-usual” scenario, in which atmospheric CO_2_ concentrations reach 700 ppm in the year 2100 (Fig. 2). Comparison of projections from the different scenarios provides a measure of physical and societal mass bleaching “commitment” given different assumptions about the adaptability of coral reef ecosystems. The results provide insight into the level of atmospheric greenhouse gas concentrations necessary to avoid degradation of coral reef ecosystems worldwide and the need for coordinated efforts to maximize coral reef resilience.

## Results

### Sea surface temperature

This section summarizes the projected change in sea surface temperatures averaged across the tropical oceans and across the eleven ocean provinces that contain coral reef ecosystems (see Materials and Methods). The simulated increase in annual mean SST for each of the coral reef provinces over the 21^st^ century ranges from lows in the Commit scenario to highs in the fossil-fuel intensive A2 and A1F1 scenarios (Table 1). The CM2.0 and CM2.1 ensemble results for the Commit scenario indicate that 0.4–0.6°C of the simulated regional SST increase by 2090–2099 in the SRES scenarios is due to the physical commitment from atmospheric greenhouse gas accumulation until the year 2000. There is an additional 0.7–0.9°C increase in regional mean SST in the B1 scenario, in which atmospheric CO_2_ concentrations stabilize at 550 ppm by 2100. The difference between the emissions path of B1 scenario and the “business-as-usual” A1b scenario results in an additional 0.8–0.9°C increase in regional mean SST.

**Table 1:** Annual mean SST anomaly averaged across each ocean province.

Region	SST anomaly 2030–2039					SST anomaly 2090–2099				
	Commit	B1	A1b	A2	A1f1	Commit	B1	A1b	A2	A1f1
Caribbean	0.4	0.8	0.9	0.9	1.1	0.6	1.5	2.4	2.7	3.4
Middle East	0.4	0.9	1.0	0.9	1.1	0.6	1.6	2.5	3.0	3.5
W Indian	0.4	0.7	0.8	0.8	1.0	0.6	1.4	2.2	2.8	3.4
C Indian	0.4	0.7	0.9	0.8	1.0	0.6	1.4	2.3	2.9	3.5
W Australia	0.3	0.8	0.9	0.8	1.0	0.5	1.3	2.1	2.8	3.4
SE Asia	0.3	0.7	0.8	0.8	0.8	0.5	1.3	2.1	2.7	3.2
GBR+Melanesia	0.4	0.6	0.8	0.8	1.0	0.5	1.2	2.1	2.7	3.3
Micronesia	0.4	0.5	0.8	0.7	1.1	0.5	1.4	2.5	3.0	3.6
Central Pacific	0.4	0.6	0.8	0.7	1.1	0.6	1.4	2.5	3.0	3.6
Polynesia	0.3	0.6	0.6	0.7	0.9	0.4	1.1	1.9	2.3	2.8
East Pacific	0.4	0.8	0.9	0.9	1.1	0.6	1.5	2.4	2.8	3.5
All tropics	**0.3**	**0.6**	**0.7**	**0.6**	**0.8**	**0.5**	**1.1**	**1.8**	**2.3**	**2.8**

The anomaly for each region is the difference between the projected CM2.0 and CM2.1 ensemble decadal mean SST models and the 1980–2000 ensemble mean SST.

**Figure 3:**
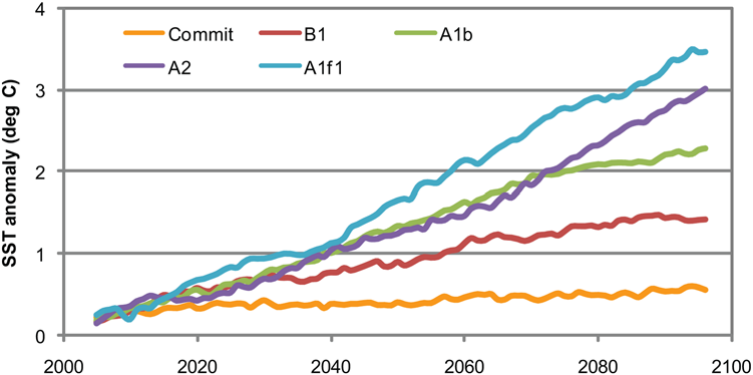
Nine-year running mean annual sea surface temperature anomaly (in °C) for the Central Indian Ocean. The anomaly is calculated from the ensemble of the CM2.0 and CM2.1 annual sea surface temperatures.

The projected SST anomalies in the different future scenarios do not diverge for several decades. The difference between the mean projected warming for 2030–2039 in the B1, A1b and A2 scenarios is ≤0.1°C for seven of the eleven reef provinces, and ≤0.3°C for the remaining four reef provinces (Table 1). For example, the nine-year running mean SST anomaly for the Central Indian Ocean reef province shows a similar trend between B1 and A1b until after the 2030s (Fig. 3). The results of the Commit scenario indicate that roughly half (0.3–0.4°C) of the 0.5–1.0°C warming projected for the reef provinces in the B1, A1b and A2 scenarios is due to the physical commitment in the climate system. The remainder of the warming by 2030–2039 is similar between the SRES scenarios because of the overlap in emissions trajectories (the “societal” commitment) in the scenarios and the lagged response of the climate system to greenhouse gases emitted during the intervening years (the “physical” commitment). The 0.5–0.9°C SST increase by 2030–2039 in the B1 scenario is a possible measure of the combined physical and societal warming commitment. A lower amount of mean surface ocean warming would require a near-term effort to reduce greenhouse gas emissions below the B1 trajectory.

### Mass Coral Bleaching

The occurrence of mass coral bleaching is commonly predicted by the accumulation of sea surface temperatures above the maximum value in the monthly climatology (e.g., the highest value in a long-term average of monthly temperatures). This baseline above which thermal stress accumulates is typically referred to as the maximum monthly mean (MMM). Here, the annual accumulation of “degree heating months” (DHM, in °C-month) above the MMM from 2001–2100 is calculated in each coral reef cell for each of the future scenarios by integrating model output with observed SST data (see Materials and Methods). A DHM value of 2°C-month is employed as an indicator of thermal stress which can lead to severe mass coral bleaching [13], [15]. The likelihood of a severe mass coral bleaching event (DHM≥2°C-month) occurring in a given year from 2005–2095 in each coral reef cell is calculated using a ten-year running period of results from both the CM2.0 and CM2.1 simulations (i.e. n_years_ = 20) for each future scenario.

For the purposes of analysis, the results are used to determine the year in which severe mass coral bleaching begins to occur more than once every five years (p = 0.2). The arbitrary five year return period represents a simple estimate of the shortest acceptable time between severe bleaching events. It is based on the minimum time required for hard coral cover to recover to pre-bleaching levels reported in the literature (e.g. [14], [18]–[21]). In reality, recovery can vary widely in space and time due to factors like coral community structure, *Symbiodinium* diversity, other stressors, and bleaching experience [21]–[26]. Recovery can also be difficult to define; even if hard coral cover returns to pre-bleaching levels, changes in the community composition and age structure may affect ecosystem function and the diversity of reef organisms [7]–[10]. A single minimum acceptable return period is applied here in order to facilitate spatial comparison of the climate projections and to avoid the model uncertainty caused by including results the field studies which used a variety of different methods and metrics to characterize recovery.

**Figure 4:**
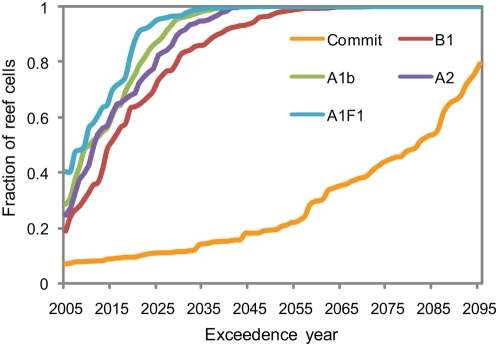
Frequency distribution of the year in which the probability of severe mass bleaching events (DHM≥2°C-month) exceeds 20% for each the 1687 coral reef cells. The probability of mass bleaching in each scenario is estimated from running 10-year intervals of both the CM2.0 and CM2.1 simulations.

The results of the Commit scenario indicate that severe coral bleaching becomes a five-year event for over half the world's coral reefs by 2080 due solely to the physical commitment from the accumulation of greenhouse gases in the atmosphere until the year 2000 (Fig. 4). The annual average DHM across each reef provinces in the different scenarios demonstrates the regional variation in current and projected frequency of thermal stress (Table 2). The results suggest the physical warming commitment poses less of a threat to reefs in the Caribbean, Middle East, Great Barrier Reef and Melanesia than to reefs in other regions. Alternatively, parts of the equatorial Pacific are expected to already experience DHM≥2°C-month almost once every five years. Coral reefs in the central and eastern equatorial Pacific experience high background SST variability due to the Southern Oscillation. The persistence of coral reefs in a region subject to frequent thermal anomalies may indicate that those ecosystems possess a naturally higher resistance to thermal anomalies or are capable of rapid recovery from thermal stress.

**Table 2:** Year that the probability of DHM>2°C-month exceeds 20%.

	Commitment		SRES B1		SRES A1b	
	Base	+1.5 K	Base	+1.5 K	Base	+1.5 K
Caribbean	n/a	n/a	2016	n/a	2018	2074
Middle East	n/a	n/a	2033	n/a	2023	2070
W Indian Ocean	2063	n/a	2024	n/a	2013	2088
C Indian Ocean	2063	n/a	2019	n/a	2012	2082
Western Australia	2071	n/a	2024	n/a	2016	2086
SE Asia	2061	n/a	2021	n/a	2012	2074
GBR+Melanesia	2095	n/a	2028	n/a	2017	2092
Micronesia	2009	n/a	2010	n/a	2005	2065
Central Pacific	2005	n/a	2005	2062	2005	2051
Polynesia	2072	n/a	2016	n/a	2015	2094
East Pacific	2048	n/a	2014	n/a	2012	2073

The results are taken from CM2.0 and CM2.1 ensemble projections under different future emissions scenarios.

In the B1 scenario, the five year frequency threshold is surpassed by 80% of the world's reefs by 2030, fifty years earlier than in the Commit scenario and ten years later than in the higher emissions A1b scenario (Fig. 4). The fraction of reefs expected to have surpassed the five year frequency threshold by 2005 increases from 7% in the Commit scenario to 19% in the B1 scenarios and 29% in the A1b scenario. This result varies between the scenarios, despite the small differences in simulated SSTs over the current decade (e.g., Fig. 3), because the projected and observed warming since the beginning of this study's 1985–2000 climatological baseline period may have already elevated the frequency of severe thermal stress close to or past the five year threshold in many locations.

**Figure 5:**
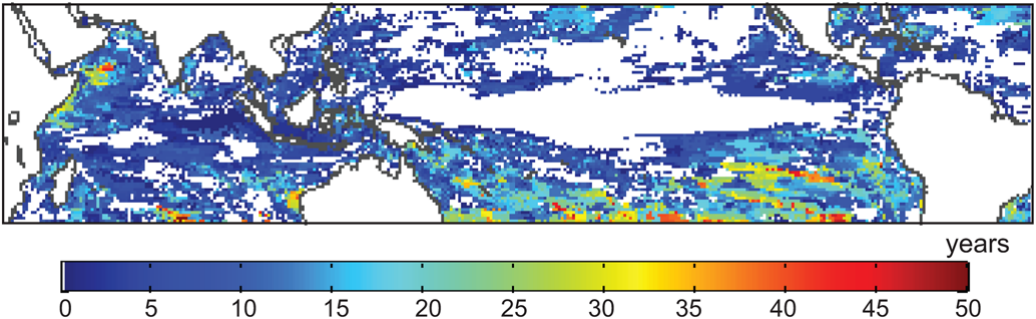
Positive difference in the exceedence year between the A1b and B1 scenarios, assuming no thermal adaptation. The exceedence year in each scenario is the ensemble mean of the year in which the probability of DHM≥2°C-month exceeds 20%. In grid cells where the probability is not reached before 2100, the difference is calculated as 2100 – exceedence year for A1b. For the purposes of presentation, results are displayed for all grid cells, rather than only coral reef cells.

The difference between the projected exceedence year in the B1 and in the A1b scenario is less than 10 years in 70% of the coral reef cells and less than 20 years in 90% of the coral reef cells. Differences of 20 years or more occur only in parts of Melanesia, Polynesia, West Australia and the Western Indian Ocean (Fig. 5). The projections are similar in the two scenarios because the combination of the physical warming commitment and the likely societal warming commitment is sufficient to regularly cause DHM accumulation in excess of the upper bleaching threshold in the next several decades. Therefore, absent any adaptation or acclimation by corals and their symbionts, the majority of the world's coral reefs could experience frequent mass bleaching events by 2030, even if the world is able to shift greenhouse gas emissions from a business-as-usual emissions path (A1b) to a 550 ppm stabilization path (B1).

### Effect of adaptation

The IPCC defined adaptation as measures that reduce the vulnerability of a system to climatic stress [12]. Using this broad definition of the term adaptation, there are several ways in which coral reefs may adapt to increasing thermal stress. Corals and their symbionts may be able to biologically adapt or acclimate to thermal stress via a range of mechanisms including symbiont shuffling and switching [27]–[30] and heterotrophic plasticity [31]. At the community level, selective coral morbidity and mortality during bleaching events may lead to dominance of more resistant and resilient growth forms and species, thus reducing vulnerability to future thermal stress events. Finally, management interventions like reducing fishing pressure and protecting thermally resistant micro-environments may be able to increase resistance to thermal stress and enhance the rate at which coral reef ecosystems recover from a mass bleaching event [32].

The effect of adaptation on the probability of severe mass bleaching events is calculated for each scenario, again using a ten-year running period of both the CM2.0 and CM2.1 simulations (n_years_ = 20). A potential increase in thermal tolerance of 1.5°C is employed, based on the outer bound of increased thermal tolerance observed in the common Indo-Pacific species *Acropora millepora* [33] and *Acropora Aspera* [34]. This value is employed as a benchmark because the genus *Acropora* encompasses one-quarter to one-third of one-quarter of scleractinian coral diversity [35], dominates shallow coral cover in parts of the Indo-Pacific [34], and is typically bleaching sensitive [23], [24], [33]. In reality, the thermal flexibility of corals should vary widely between species, growth forms and environments. The assumption of a 1.5°C thermal flexibility is a best guess of what could be achieved by some common corals through biological mechanisms and management efforts based on existing literature.

**Figure 6:**
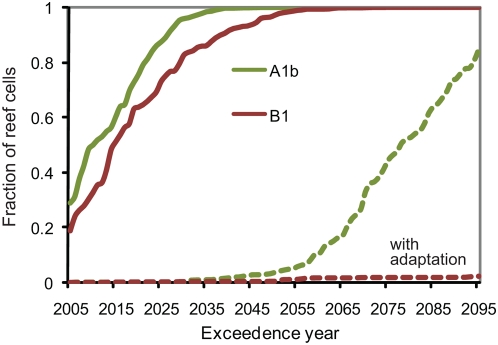
Frequency distribution of the year in which the probability of severe mass bleaching events (DHM≥2°C-month) exceeds 20% for each the 1687 coral reef cells. The frequency of mass bleaching in each scenario is estimated from running 10-year intervals of both the CM2.0 and CM2.1 simulations. Shown are results for SRES A1b and SRES B1, assuming no thermal adaptation (thick lines) and 1.5°C thermal adaptation (dashed lines).

A 1.5°C increase in thermal tolerance postpones the passage of the five year return period until at least the latter half of the century for almost all of the world's coral reefs in each scenario (Fig. 6). No coral reef cells pass the threshold before 2050 in the Commit scenario or the B1 scenario, and only 4% pass the threshold before 2050 in the A1b scenario. By the end of the century, over 80% of the coral reef cells experience bleaching at least once every five years in the A1b scenario. In the B1 scenario, however, less than 3% of coral reef cells experience bleaching once every five years by the end of the century. Examining the results by region, only the Central Pacific passes the five year bleaching threshold during this century in the B1 scenario (Table 2). The increase in thermal tolerance delays the bleaching threshold the longest in regions like Polynesia, where the models project lower warming relative to the global mean warming, as in [15].

The divergence between A1b and B1 bleaching trajectories (Fig. 6) in the adaptation case occurs essentially because the increase in thermal tolerance exceeds the total physical and societal warming commitment, roughly depicted by the B1 scenario, but not the additional warming expected under the business-as-usual A1b scenario. The difference between the trajectories suggest sustained 1.5°C increase in the thermal tolerance of corals and their symbionts would, at minimum, postpone severe mass bleaching from becoming a five-year event for the majority of the world's reefs until the latter half of the century. If, in addition to the sustained increase in thermal tolerance, mitigation efforts shift atmospheric CO_2_ concentrations from the “business-as-usual” trajectory depicted in the A1b scenario to the 550 ppm stabilization trajectory depicted in the B1 scenario, the models results indicate that severe mass bleaching would become a five-year event for a small minority (<3%) of the world's coral reefs this century.

The analysis of climate change impacts under future emissions scenarios often ends in the year 2100, even though the climate system will not have reached equilibrium. Residual warming would occur after the stabilization of greenhouse gas concentrations in the year 2100 in the GFDL simulations of the A1b and B1 scenarios (similar to the committed warming during this century in the Commit scenario). In the B1 scenario, for which the model runs extended beyond 2100, residual warming could cause 11–22% of the world's coral reefs to experience mass bleaching events once every five years by the end of the 22^nd^ century.

### Sensitivity analysis

This section analyzes the sensitivity of the future projections to method of the bleaching prediction. The thermal stress projections are repeated using two proposed but globally untested changes to the real-time method of bleaching prediction: i) introduction of a bleaching threshold based on historical SST variability, and ii) use of a different formulation for the maximum monthly mean.

#### i) Incorporation of historical SST variability

Corals exposed to higher background variability in maximum annual SST and more frequent thermal anomalies may have developed a higher thermal resistance [25], [36]. The common metric for predicting the onset of coral bleaching – one month with SSTs that are 1°C greater than the maximum monthly mean (MMM) – is a description of a low frequency event in the majority of the tropical ocean. In regions with high year to year background SST variability, however, the 1°C threshold represents a higher frequency event. A metric based on the inter-annual variability in maximum monthly SST may be a better predictor of the onset of mass coral bleaching in low or high variability regions.

The effect of background SST variability on the results of this study is evaluated by repeating the future projections using a bleaching metric based on the standard deviation (σ_M_) of the SSTs from the climatological maximum month in the 1985–2000 satellite climatology. The values of σ_M_ for the coral reef cells around the world range from 0.15°C to 1.32°C, with a 5^th^ to 95^th^ percentile range from 0.26°C to 0.64°C, and a median of 0.41°C. A median global average bleaching threshold of 1°C in excess of the maximum monthly mean SST is equivalent to 2.45σ_M_; the severe bleaching threshold of 2°C used in this study is equivalent to 4.90σ_M_. The SST variability-based metric is incorporated into the bleaching projections by replacing the DHM>2°C-month threshold for the occurrence of severe mass bleaching is replaced with DHM>4.90σ_M_ °C-month.

**Figure 7:**
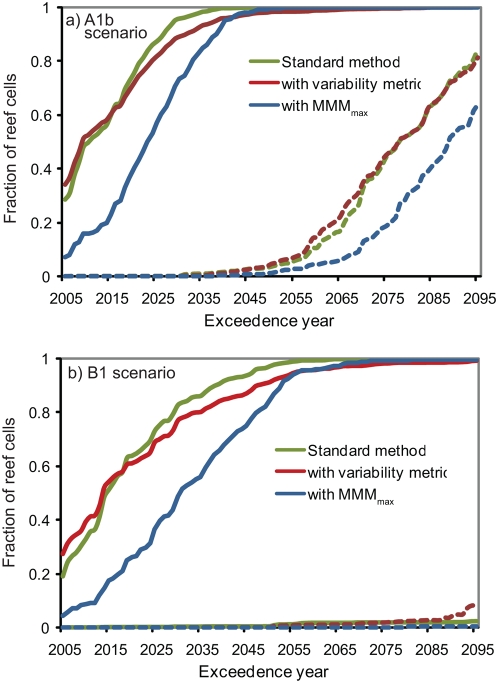
Frequency distribution of the year in which the probability of severe mass bleaching events (DHM≥2°C-month) exceeds 20% for each the 1687 coral reef cells from the sensitivity analysis. Shown are results for a) the A1b and b) the B1 scenario assuming no thermal adaptation (thick lines) and 1.5°C thermal adaptation (dashed lines).

The variability-based threshold has a normalizing effect on the global results, delaying the bleaching projection for coral reef cells with high background SST variability and advancing the bleaching projection for coral reef cells with low background SST variability (Fig. 7). The year in which severe bleaching becomes a five-year event is delayed in 32% (34%) of the coral reef grid cells in the A1b (B1) scenario if the variability-based threshold is used instead of the standard bleaching threshold (Table 3). The occurrence of thermal anomalies is more sensitive to the bleaching prediction algorithm in the lower emissions B1 scenario due to the slower rate of SST increase in most regions. In the majority of the cases in which the variability-based thresholds delays the occurrence of the five year bleaching frequency, the delay is by less than 10 years. The year that mass bleaching becomes a five year event is delayed by 10 years or more in 11% (18%) of all coral reefs cells in the A1b (B1) scenario, and 20 years or more in 4% (9%) in the A1b (B1) scenario. The largest delays in the bleaching projections occur for coral reefs in Western Australia, the Middle East and the Eastern Pacific.

**Table 3:** Fraction of coral reef cells in which the projected year that the probability of severe bleaching exceeds 20% is changed by incorporating alternate formulations of the bleaching algorithm.

Change in projection (yr)	SST variability^1^		MMM_max_ 2	
	A1b	B1	A1b	B1
<20 yr earlier	-	2%	-	-
−19 to −10 yr	6%	9%	-	-
−1 to −9 yr	31%	26%	-	-
No change	37%	32%	13%	10%
1 to 9 yr later	20%	16%	47%	29%
+10 to +19 yr	6%	8%	29%	35%
+20 to +29 yr	2%	4%	11%	19%
+>30 yr	1%	4%	1%	6%

1 SST variability refers to a severe bleaching threshold based on historical SST variability.

2 MMM_max_ refers to the mean of the maximum monthly SST from each year in place of the MMM from the 1985–2000 SST climatology.

Conversely, the year in which severe bleaching becomes a five-year event is advanced in 34% (37%) of the coral reef grid cells in the A1b (B1) scenario by incorporation of the variability-based threshold (Table 3). The bleaching projections are advanced for coral reefs in the centre of the low variability West Pacific Warm Pool, which includes the parts of Micronesia, Melanesia and South East Asia, as well as some coral reefs in Polynesia, the western and Central Indian Ocean and the Caribbean. The projected year that the five year frequency threshold is surpassed is less than 10 years early in 91% (70%) of these cases in the A1b (B1) scenario, and more than 20 years earlier in 5% of these cases in the B1 scenario.

It should be noted that the accuracy of the variability-based bleaching metric is limited by available data. While there is evidence that background SST variability influences thermal sensitivity of coral communities [25], there are few well-documented observations of mass coral bleaching in the remote regions with extremely low background SST variability (e.g., Micronesian islands in the West Pacific Warm Pool [37]) and extremely high background SST variability (e.g. central equatorial Pacific Atolls affected by ENSO). Further observational data in these regions is necessary to conduct proper statistical tests of the metric and evaluation of lower and upper bounds for the DHM thresholds. For example, since the variability-based metric as constructed does not assign a minimum value to the DHM threshold, the long tail of extremely low σ_M_ values increases the fraction of reefs expected to have surpassed the five year frequency threshold by 2005 to 34% (27%) from 29% (19%) in the A1b (B1) scenario.

#### ii. Alternate MMM

The method of calculating the MMM can bias the prediction of thermal anomalies. The MMM is intended to represent the average maximum SST experienced by corals and their symbionts each year. In equatorial waters with typically low SST seasonality, inter-annual climate oscillations like the El Nino/Southern Oscillation influence the timing of the annual peak in SST. For example, in the central equatorial Pacific atoll of Kiritimati, the maximum annual SST occurred in eight different months between 1985 and 2000 [38]. In such locations, the maximum SST in the monthly climatology is lower than a mean of the maximum monthly SST experienced each year. Therefore, the use of a climatological MMM could overestimate the frequency and magnitude of bleaching-level thermal stress at some equatorial reefs.

The future projections are repeated using the mean of the maximum monthly SST of each year from 1985 to 2000 (MMM_max_) as the MMM, rather than the maximum from the monthly SST climatology. The difference between MMM_max_ and MMM for the coral reef grid cells ranges from 0°C to 0.82°C, with a 5^th^ to 95^th^ percentile range from 0.05°C to 0.39°C, and a median of 0.20°C. The distribution of MMM_max_ - MMM is positively skewed (skewness = 0.71) indicating a long tail of high values, mostly located in the equatorial Pacific.

The year in which severe bleaching becomes a five-year event is “delayed” in 87% (90%) of the coral reef grid cells in the A1b (B1) scenario if the MMM_max_ is used in place of the MMM (Table 3). The median delay is 8 (13) years in the A1b (B1) scenario. The delay is greater than 20 years in only 12% (25%) of coral reef cells in the A1b (B1) scenario. The largest differences (maximum is 34 years in A1b, 78 years in B1) occur in Micronesia, Melanesia, the Philippines and other regions where although there is low seasonality to SSTs in the 1985–2000 climatology, the annual timing of the SST peak varied due to the migration of the West Pacific Warm Pool and to the El Nino/Southern Oscillation.

Globally, substituting MMM_max_ for the MMM has a larger effect on the future projections than using a bleaching threshold based on historical SST variability (Fig. 7). For example, in the A1b scenario without thermal adaptation, the point at which severe mass bleaching becomes a five year event for 50% of the coral reef cells is delayed on average by 12 years by the use of MMM_max_, and is advanced by two years by the use of the variability-based bleaching threshold. The difference is of a similar order in the B1 scenario, with and without the thermal adaptation assumption. Although applying the alternative formulations alters the exceedence years for many coral reef cells, it does not alter the general finding that without any adaptation the physical and societal warming commitment depicted by the B1 scenario would sufficient to cause the majority (>98%) of the world's coral reefs to experience bleaching events at least once every five years by the end of this century.

## Discussion

This analysis finds that the committed warming from the accumulation of greenhouse gases in the atmosphere up until the year 2000 is alone sufficient to cause the majority of the world's coral reefs to experience harmfully frequent thermal stress events by the end of the century (Fig. 4). A possible additional warming commitment caused by the time required to shift from a “business-as-usual” emissions trajectory to a 550 ppm CO_2_ “stabilization” trajectory may cause the majority of the world's coral reefs to experience harmfully frequent thermal stress events before the year 2050. A 1.5°C increase in the thermal tolerance of corals and their symbionts would postpone the “business-as-usual” severe bleaching forecast by 50–80 years for most of the world's coral reefs. The increase in thermal tolerance could provide time to shift greenhouse gas emissions to the 550 ppm stabilization trajectory and avoid the recurrence of harmfully frequent severe coral bleaching events at majority (∼97%) of the world's coral reefs during this century. The physical warming commitment from the accumulation of atmospheric greenhouse gas concentrations in the B1 stabilization scenario would however cause a further increase in the frequency of thermal anomalies beyond 2100.

The sensitivity analysis indicates that the global projections are not highly sensitive to the prediction method. Application of the variability-based bleaching threshold advances the projections for coral reefs in the Micronesia, Melanesia, SE Asia and selected other locations, and delays the projections for coral reefs in the eastern Pacific, the Middle East, and Western Australia by several decades. However, it has a small effect when averaged globally. Introduction of MMM_max_ delays the global bleaching projections by 10–12 years, with the largest effect in low seasonality regions like the western and central equatorial Pacific. These two alternative prediction methods are currently experimental and require further development and evaluation before being fully integrated into real-time forecasting. Recent research indicates the variability-based bleaching threshold will more accurately predict the onset of mass bleaching than a globally fixed threshold [25], [26], [37]; however, further evaluation of this metric is necessary in regions with extreme low and high background SST variability. The MMM_max_ approach may be most appropriate for select regions like the central equatorial Pacific [38] where high year-to-year variability in timing of peak SST complicates calculation of a baseline temperature above which thermal stress accumulates. While neither alternative prediction method alters the nature of the global forecast presented here, they do reveal regions where climatic experience could confer greater sensitivity or greater resistance to future climate change.

The overall results of this study can provide insight into the level of atmospheric greenhouse gas concentrations required to avoid degradation of coral reef ecosystems from frequent mass coral bleaching, a proposed definition of “dangerous anthropogenic interference” in the climate system [39]. Specific recommendations about future greenhouse gas emissions pathways and/or atmospheric stabilization levels require normative judgements about the acceptable damages to coral reefs and the metrics for characterizing those damages. A comparison of the results from the key scenarios in this study does, however, present an envelope of possible climate futures for the world's coral reefs, presuming that the models realistically represent the response of the climate system to external forcing.

At one extreme, in which there is no sustained thermal adaptation, atmospheric CO_2_ concentrations would need to be stabilized below 370 ppm, the global mean mixing ratio for the year 2000, to avoid degradation of coral reef ecosystems. A recent paleoclimate analysis recommended stabilization of atmospheric CO_2_ at a similar level (350 ppm) to avoid large-scale climate disruption [40]. In this case, the long-term function of coral reef ecosystems would depend on the ecosystem's ability recover from damages sustained by warming caused by the “overshoot” of the stabilization target. At the other extreme, in which thermal adaptation of 1.5°C occurs with minimal cost to reef function and diversity, atmospheric CO_2_ concentrations would need to be stabilized at a maximum of 550 ppm by the year 2100.

The results of research on ocean acidification, the sensitivity analysis and the aggressive assumptions about adaptation taken in this study suggest a CO_2_ stabilization target at the upper end of the indicated range would likely fail to avoid degradation of most coral reef ecosystems. First, the absolute upper bound for atmospheric CO_2_ stabilization is higher than the 450–500 ppm range recommended to avoid deleterious effects of rising surface ocean pCO_2_ on reef accretion [4], [41]. For some reef species, elevated pCO_2_ could act synergistically with warmer SSTs and effectively decrease thermal tolerance, although an offsetting effect may also occur [42]. Second, the bleaching projections calculated with the variability-based bleaching threshold indicate that coral reefs in regions of low background SST variability could be more sensitive to increasing SST than reported in previous studies [15]. If so, a CO_2_ stabilization target at the upper end of the range would fail to protect many coral reefs in low variability regions like SE Asia, home to highest scleractinian coral diversity [35].

Finally, the assumptions about the rate of recovery from coral bleaching events and the potential range of thermal adaptation taken in this study are highly idealized. Even in the optimistic scenario where total hard coral cover on reefs worldwide can be restored to pre-bleaching levels in five years, there are likely to be declines in diversity and biomass of corals, coral reef fish and other reef organisms [7]–[11], [43]. In addition, even if the proposed increase in thermal tolerance is biologically possible, it will require time to fully take effect (e.g., for temperature-tolerant *Symbiodinium* and/or more thermally resistant coral taxa to become dominant). These biological changes will also come with costs including reduced productivity and fecundity in surviving corals [44], [45] and regional or global extinction of bleaching-sensitive coral species [43]. Coral morbidity and mortality may also occur due to disease outbreaks associated with thermal stress [46]. Taken together, these effects are likely to reduce the ability of surviving corals to compete with fleshy plants and other organisms for the substrate.

In summary, the results of this study indicate that a combination of greenhouse gas mitigation and improved coral reef management will be required to avoid the degradation of the world's coral reef ecosystems from frequent mass coral bleaching events. Actions that enhance reef resistance and reef resilience - including protection of bleaching-resistant reefs, reduction of other stressors, and possibly even more radical suggestions like “seeding” reefs with more temperature-tolerant species of *Symbiodinium* – may be necessary to help coral reef ecosystems endure through the committed warming over the next several decades. These management actions, while important, will alone prove to be insufficient to protect coral reefs through the latter half of the century. The difference between the future scenarios presented in this study demonstrates that protecting the world's coral reefs from increasing thermal stress will require a dramatic reduction in greenhouse gas emissions over the next several decades.

## Materials and Methods

### Input data

Simulated sea surface temperatures (SSTs) are obtained from simulations of the coupled atmosphere-ocean general circulation models (GCM) CM2.0 and CM2.1 conducted for the IPCC's Fourth Assessment Report (FAR). Previous global assessments of coral bleaching under future climate change employed model simulations from earlier IPCC assessments [1], [15]. These particular GCMs provide two key advantages for coral reef research over other GCMs. First, the climate sensitivities of CM2.0 and CM2.1 are 2.9°C and 3.4°C respectively are in the middle of the 2.1°C–4.4°C range of climate sensitivities (mean value = 3.2°C) of models used in the FAR [47]. The difference in the climate sensitivities of the two models is caused by assumptions in the dynamic core of the atmospheric component of the otherwise similar models. Taken together, the output of CM2.0 and CM2.1 represents a median estimate from FAR.

Second, the ocean component of CM2.0 and CM2.1 operates on a higher resolution grid than most other GCMs. The grid has a longitudinal resolution of 1° and a latitudinal resolution ranging from 1° in the mid-latitudes (30° north and south) to 1/3° at the equator [17]. The similarity between the models' resolution in the tropics and that of the available satellite-derived SST data eliminates the need for statistical downscaling of model output to the resolution of the satellite data. It also reduces the conflict between coastal geography in the GCM and the satellite map. The higher model resolution is particularly critical for research on tropical coastal ecosystems because a large proportion of more closed ocean basins like the Coral Triangle in Southeast Asia and the Caribbean are represented as land in lower resolution GCMs [15].

The simulated SSTs from historical “all-forcing” simulations for the 1870–2000 period and five different future emissions scenarios for the 2001–2200 period are used in this study. The “all-forcing” simulations (CM2.0, n = 3; CM2.1, n = 5), which simulate historical climate variability based on forcings of greenhouse gases, sulfate and volcanic aerosols, black and organic carbon and solar irradiance, are used to develop a model climatology (see below). The “commitment” scenario, in which greenhouse gas concentrations are stabilized in the year 2000, represents the physical warming due to thermal inertia of the climate system (Fig. 2). In reality, continued emissions growth of 2–3 ppm CO_2_ per year raised the mean atmospheric CO_2_ concentration from the 370 ppm in 2000 to 383 ppm in 2007. Therefore, stabilization at the level in the commitment scenario would require some net drawdown of atmospheric CO_2_ via sequestration.

The four SRES scenarios (B1, A1b, A2, A1F1) represent different describe different emissions paths (Fig. 1). The B1 scenario describes atmospheric CO_2_ concentrations stabilizing at 550 ppm in 2100 due to a reduction in emissions rate below today's levels. Alternatively, the A1b describes a “business-as-usual” emissions path, resulting in atmospheric CO_2_ concentrations reaching 700 ppm in 2100. The A2 and A1F1 scenarios describe a future more dependent on fossil fuels than the other scenarios.

A 0.5°×0.5° map of coral reefs is derived from Reefbase (see http://www.reefbase.org) and manual corrections (Fig. 1). The map features 2157 grid cells containing some area of warm-water coral reef. The conflict between the land mask of CM2.0 and CM2.1 and the land mask of the satellite data, primarily in Southeast Asia and the Middle East, reduces the number of coral reef cells analyzed in this study by 470 (22%) to 1687 grid cells. The coral reef provinces are defined based on the ocean basins, recognized patterns in coral species diversity and major oceanographic features like the typical reach of the El Nino/Southern Oscillation, as in previous studies [15], [37]. The few physically isolated coral reef grid cells not located within the defined reef provinces (3% of cells) are still included in the global analysis.

### Estimating thermal stress

Historical monthly sea surface temperature (SST) data for 1985–2005 is derived from the 0.5×0.5 degree resolution AVHRR Pathfinder dataset used by the NOAA Coral Reef Watch program to predict coral bleaching in real-time [48]–[50]. This biweekly data is based on a retrospective analysis that accounts for sensor drift and atmospheric attenuation through comparisons of satellite-derived data with in-situ data from NOAA's network of buoys. The NOAA Coral Reef Watch program uses the data to issue real-time coral bleaching alerts, based on the weekly accumulation of temperatures in excess of a climatological maximum [48]–[50].

Monthly SSTs are presented as the sum of the satellite-derived monthly climatology and model anomalies. For example, the SST for March, 2030 in a given scenario is the sum of the March SST in the satellite climatology and the difference between the simulated SST for March, 2030 and the simulated climatological SST for March. The climatological period used in coral bleaching prediction is typically a minimum of seven years between 1985 and 2000 [13], [15], [48]–[51]. Here, the full 1985–2000 period is used as the climatology to maximize statistical agreement between the observed climatology and the model climatology [13].

The thermal stress on corals is estimated using the accumulation of “degree heating months” (DHMs) over a four month rolling window. One DHM (in °C-month) is equal to one month of SST that is 1°C greater than the maximum in the monthly climatology, known as the maximum monthly mean (MMM). The monthly time step is better suited to the temporally coarse archived GCM output than weekly time step used in real-time prediction [51]. Previous analyses concluded that bleaching typically begins (NOAA Bleaching Alert 1) at DHM>1°C-month and becomes severe and mortality is observed (NOAA Bleaching Alert 2) at DHM>2°C-month [15]. Using this method, the simulated annual accumulation of DHM for a given year in a given emissions scenario is equal to the maximum four-month accumulation of simulated SST (model anomaly plus satellite climatology) in excess of the maximum monthly mean from the 1985–2000 satellite climatology [13].

The results are presented in terms of probabilities or frequencies of DHM values in excess of the upper bleaching threshold. GCMs are best suited to describe the evolution of the statistical properties of the climate system over time (e.g. bleaching events per decade), rather than the specific state of the climate at a particular moment in the future (e.g., bleaching event occurs on Jan 31, 2036). Therefore, studies of the effect of climate change on discrete events like mass coral bleaching or heat waves typically translate the time series of occurrences of the events into a time series of frequencies or probabilities [13], [52].
